# Nonresident parental influence on adolescent weight and weight-related behaviors: similar or different from resident parental influence?

**DOI:** 10.1186/s12966-014-0131-y

**Published:** 2014-10-23

**Authors:** Jerica M Berge, Craig Meyer, Richard F MacLehose, Marla E Eisenberg, Dianne Neumark-Sztainer

**Affiliations:** Department of Family Medicine and Community Health, University of Minnesota Medical School, Phillips Wangensteen Building, 516 Delaware Street SE, Minneapolis, MN 55455 USA; Division of Epidemiology and Community Health, University of Minnesota, Phillips Wangensteen Building, 516 Delaware Street SE, Minneapolis, MN 55455 USA; Division of Adolescent Health and Medicine, University of Minnesota, Phillips Wangensteen Building, 516 Delaware Street SE, Minneapolis, MN 55455 USA

**Keywords:** Resident parent, Nonresident parent, Adolescents, Obesity, Dietary intake, Physical activity

## Abstract

**Background:**

Many parents do not live with, or have shared custody of, their adolescent children (i.e., nonresident parents). The degree of their influence on their children, as compared to parents who do live with their children the majority of the time (i.e. resident parents) has not been well-studied. The current study aimed to examine whether and how resident and nonresident parents’ weight and weight-related behaviors are correlated with adolescents’ weight and weight-related behaviors. Results will inform who may be important to include in adolescent obesity prevention interventions.

**Methods:**

Data from two linked population-based studies, EAT 2010 and F-EAT, were used for cross-sectional analyses. Resident parents (n = 200; 80% females; mean age =41.8), nonresident parents (n =200; 70% male; mean age =43.1), and adolescents (n =200; 60% girls; mean age =14.2 years) were socioeconomically and racially/ethnically diverse. Multiple regression models were fit to investigate the association between resident and nonresident parents’ weight and weight-related behaviors and adolescents’ weight and weight-related behaviors.

**Results:**

Both resident and nonresident parents’ BMI were significantly associated with adolescents’ BMI percentile. Additionally, resident parents’ sugar-sweetened beverage consumption and fruit and vegetable intake were significantly associated with adolescents’ sugar-sweetened beverage intake and fruit and vegetable intake (p < 0.05), respectively. Furthermore, the association between nonresident parent physical activity and adolescent physical activity was marginally significant (p = 0.067). Neither resident nor nonresident parents’ fast food consumption, breakfast frequency, or sedentary behaviors were significantly associated with adolescents’ same behaviors.

**Conclusions:**

These preliminary findings suggest that resident and nonresident parents may have slightly different influences on their adolescent children’s weight-related behaviors. Longitudinal follow-up is needed to determine temporality of associations.

## Introduction

According to national data, it is common for adolescents to live in shared custody arrangements [1]. In fact, 40% of adolescents from divorced or separated families live in households where custody is equally shared and 50% live in households where some combination of custody sharing is occurring (only 10% have no shared custody arrangements) [1]. Few studies have examined the role of nonresident parents (i.e., child lives with parent <50% of the time) in adolescents’ weight and weight-related behaviors (e.g., fruit and vegetable intake, physical activity behaviors, dieting) [2]. Understanding whether and how resident (i.e., child lives with parent ≥50% of the time) and nonresident parents’ weight and weight-related behaviors are associated with adolescents’ weight and weight-related behaviors will inform researchers regarding which family member(s) may be important to include, or focus on, in family-based obesity prevention interventions.

The one study that has examined nonresident parental influence on adolescent eating patterns showed that adolescents in nontraditional households (i.e., single parent homes) ate significantly less healthfully than their counterparts living in dual-headed households [2]. However, adolescents in single-headed households who had nonresident father involvement had more healthful eating patterns than adolescents in single-headed households without nonresident father involvement. Thus, nonresident parents may potentially have a positive influence on adolescent weight-related behaviors.

We are aware of only one study that has examined the differential influence of resident and nonresident parents’ influence on adolescent weight and weight-related behaviors (described above) [2]. However, several studies have investigated primary and secondary parent influences within the same home environment in relation to adolescent weight and health behaviors [3-5]. Specifically, parenting style [4,6,7], parent modeling and encouraging of health behaviors [8], conversations about weight and weight-related behaviors [9], parent feeding practices [10-12], and frequency of family meals [13,14] have all been examined to identify risk and protective factors for adolescent obesity risk in the home environment [3,14]. Numerous studies have indicated that primary caregivers’ (primarily mothers) parenting style [3,7,15], feeding practices [10,11,13] and modeling and encouraging of healthful behaviors [8,16,17] were more strongly associated with adolescents’ BMI z-score, dietary patterns, physical activity habits and unhealthy weight control behaviors as compared to secondary parents (primarily fathers), although not all associations have been consistent across studies. A few studies have shown that secondary caregivers’ parenting style, feeding practices, modeling and encouraging of healthful behaviors were equally or more strongly associated with adolescents’ BMI z-score, dietary patterns, or physical activity habits as compared to the primary caregiver (e.g., father parenting style was significantly associated with more fruit and vegetable intake and lower BMI in daughters) [4,6]. Thus, it is important to continue to examine differential parental influences on adolescents’ weight and weight-related behaviors and specifically nonresident parental influences since there has been little research conducted with nonresident parents. Additionally, it is important to know for adolescent obesity intervention research whether resident and nonresident parental influence on adolescent weight and weight-related behaviors is similar or different in order to target key people in adolescents’ lives that may have the highest likelihood of impacting adolescents’ weight-related behaviors.

Thus, given the increased prevalence of adolescent obesity in the last three decades [18], the negative health consequences associated with youth obesity [19], and the frequency of shared custody arrangements of adolescents [1], it is important to know whether and how resident and nonresident parents’ weight and weight-related behaviors (i.e., BMI, fruit and vegetable intake, sugar-sweetened beverage consumption, breakfast frequency, fast food intake, dieting behavior, physical activity, sedentary behaviors) are associated with their adolescents’ weight and weight-related behaviors. The main guiding hypothesis of the study is that resident parents’ weight and weight-related behaviors will be more strongly associated with adolescents’ weight and weight-related behaviors as compared to nonresident parents’ weight and weight-related behaviors.

## Methods

### Study design and population

Data for this analysis were drawn from two coordinated, population-based studies [9]. EAT 2010 (Eating and Activity in Teens) was designed to examine dietary intake, physical activity, weight control behaviors, weight status and factors associated with these outcomes in adolescents. Project F-EAT (Families and Eating and Activity Among Teens) was designed to examine factors within the family and home environment (e.g., parent behaviors, family functioning, home food and physical activity resources) of potential relevance to adolescents’ weight and weight-related behaviors [20]. Survey development for both EAT 2010 and F-EAT are described elsewhere. Drafts of the surveys were pre-tested by 56 adolescents and 35 parents from diverse backgrounds for clarity, readability and relevance; and reviewed by an interdisciplinary team of experts. After revisions, the survey was additionally pilot tested with a different sample of 129 middle school and high school students and 102 parents to examine the test-retest reliability of measures over a one-week period. All study procedures were approved by the University of Minnesota’s Institutional Review Board Human Subjects Committee and the participating school districts.

For EAT 2010, surveys and anthropometric measures were completed by 2,793 adolescents from 20 public middle schools and high schools in the Minneapolis/St. Paul metropolitan area of Minnesota during the 2009–2010 academic year. The mean age of the study population was 14.4 years (SD = 2.0) and adolescents were equally divided by gender (47% boys, 53% girls). The racial/ethnic backgrounds of the participants were as follows: 19% white, 29% African American or Black, 20% Asian American, 17% Hispanic, 4% Native American, and 11% mixed or other. The socioeconomic status (SES) of participants included: 29% low SES, 24% low-middle SES, 33% Middle SES, 6% Upper-Middle SES, and 3% High SES.

For Project F-EAT, data were collected by surveying up to two parents/caregivers (n = 3,709) of the adolescents in EAT 2010 by mail or phone interviews. In total, 2,382 EAT 2010 (85%) adolescent participants had at least one parent respond and there were two parent respondents for 1,327 adolescents. Parent participants had a mean age of 42.3 years (SD = 8.6). The majority of parent respondents were mothers or other female parents (62%). Participating parents of adolescents were ethnically and socioeconomically diverse, similar to the adolescent sample.

The study population for the current analysis was restricted to those adolescents (n = 200) with two parent respondents, with one parent reporting that their child spent more time living with them (i.e., resident parent; lives with child ≥50% of the time) compared to the other parent (i.e., nonresident parent; lives with child <50% of the time). As no gold standard exists in the literature to determine resident vs. nonresident parent status, these cutpoints were established under the premise that a child living with one parent more than the other, at least half of the time, represented resident status. Adolescent participants in this study were predominantly female (60%), African-American (31%), with a mean age of 14.2 years. Resident parents were 80% female, had a mean age of 41.8 years, were 34% white, and 24% finished college or more. Nonresident parents were 70% male, had a mean age of 43.1 years, were 37% African American, and 19% finished college or more (See Table 1).

**Table 1:** **Demographics for adolescents, resident parents (lives with child ≥50**% **of the time) and nonresident parents (lives with child <50**% **of the time)**

**Adolescent**	**2010 (n = 200)**
Female	60% (120)*
Age	14.2 (1.86)
White	23% (45)
African American	31% (61)
Hispanic	9% (19)
Asian American	18% (36)
Hawaiian/Pacific Islander/Other	19% (39)
**Resident parent**	
Female	80% (160)
Age	41.8 (8.0)
Finished college or More (e.g. MD, PhD)	24% (48)
White	34% (67)
African American	31% (61)
Hispanic	10% (19)
Asian American	18% (35)
Hawaiian/Pacific Islander/Other	7% (14)
**Non-resident parent**	
Male	70% (140)
Age (years)	43.1 (9.5)
Finished college or More (e.g. MD, PhD)	19% (38)
White	28% (55)
African American	37% (74)
Hispanic	6% (13)
Asian American	18% (35)
Hawaiian/Pacific Islander/Other	9% (18)

*Values presented as mean (standard deviation) or % (n).

### Measures

#### BMI

*Parent Body Mass Index (BMI)* was assessed using parent self-report of height and weight (Test-retest r = .97). Self-reported height and weight has been shown to be highly correlated with objectively measured values in adults [21]. BMI was calculated using the standard formula, weight (kg)/height (meters)^2^. *Adolescent BMI* was measured by taking students’ heights and weights at school by trained research staff in a private area with standardized equipment and procedures. Adolescents were asked to remove shoes and outerwear (e.g., heavy sweaters). BMI z-scores were calculated according to the following formula: weight (kg)/height (meters)^2^ and converted to z-scores, standardized for gender and age [22,23].

#### Dietary intake

*Parent fruit and vegetable intake* was assessed by asking parents the following two questions, based on a reliable/valid measure used in a previous study [24,25], “Thinking back over the past week, how many servings of fruit did you usually eat on a typical day? (A serving is a half cup of fruit or 100% fruit juice, or a medium piece of fruit)” and “Thinking back over the past week, how many servings of vegetables did you usually eat on a typical day? (A serving is half a cup of cooked vegetables or one cup of raw vegetables)”. For both items there were seven response options (0, <1, 1, 2, 3, 4, 5 or more servings/day) (Test-retest r = 0.69 [fruit]; r = 0.57 [vegetables]). Responses for fruit and vegetable intake were coded numerically as 0, 0.5, 1, 2, 3, 4, and 5.5, then summed together to create one variable*. Parent sugar-sweetened beverage consumption* was assessed by asking parents, “Thinking back over the past week, how often did you drink sugar-sweetened beverages (regular soda, pop, Kool-Aid)?” Response options included: less than once per week, 1 drink per week, 2–4 drinks per week, 5–6 drinks per week, 1 per day, or 2 or more per day (Test-retest =0.66). Responses were coded numerically as 0, 1.5, 3, 5.5, 7 and 14, then divided by 7 for daily sugary beverage intake.

*Adolescent dietary intake* was assessed with the 149-item Youth and Adolescent Food Frequency Questionnaire (YAQ) [26]. For fruit and vegetable intake, daily servings were defined as the equivalent of one-half cup. A serving of sugar-sweetened beverages (e.g. soda pop, sports drinks) was defined as the equivalent of one glass, bottle, or can. Validity and reliability of the YAQ have been previously tested with youth and found to be within acceptable ranges for dietary assessment tools [26,27]. Responses to questions on the frequency of intake of fruits (n = 14; excluding fruit juice) and vegetables (n = 20; excluding french fries), were summed to assess average total daily intake.

#### Fast food intake

*Parent and adolescent fast food intake* was assessed by asking: “In the past week, how often did you eat something from a fast food restaurant, such as McDonald’s, Burger King, Domino’s or similar places (pizza counts)?” There were six response options: 0, 1–2, 3–4, 5–6, 7 or more than 7 times/week (Test-retest r = 0.55 [parent]; r = 0.38 [adolescent]). Responses were coded numerically as 0, 1.5, 3.5, 5.5, 7 and 9.

#### Dieting

*Parent and adolescent dieting* was assessed by self-report using the following question, “How often have you gone on a diet during the last year? By ‘diet’ we mean changing the way you eat so you can lose weight.” [28] Responses included: never, one to four times, five to 10 times, more than 10 times, and I am always dieting (Test-retest =0.60 [parent]; r = 0.65 [adolescent]). To distinguish dieters from non-dieters, responses were dichotomized into no (never) and yes (other responses). Sensitivity analyses indicated that collapsing the dieting variable produced similar results as the original 5-item scale.

#### Physical activity

*Parent and adolescent physical activity* questions were adapted from the Godin Leisure-Time Exercise Questionnaire [29]. parents and adolescents were asked: “In a usual week, how many hours do you spend doing the following activities: (1) strenuous exercise (e.g. biking fast, aerobics, jogging, basketball, swimming laps, soccer, rollerblading) (2) moderate exercise (e.g. walking quickly, easy bicycling, volleyball, skiing, dancing, skateboarding, snowboarding).” Response options ranged from “none” to “6+ hours a week”. (Test-retest r =0.75 [parent]; r = 0.72 [adolescent]). Items were summed together to assess average hours of moderate and vigorous physical activity per week.

#### Sedentary behavior

*Parent and adolescent sedentary behavior* was assessed by asking parents, “In your free time on an average weekday (Monday-Friday), how many hours do you spend doing the following activities?…[0 hr, ½ hr, 1 hr, 2 hr, 3 hr, 4 hr, 5+ hr].” [30] The activities assessed included: Watching TV/DVDs/videos, Using a computer (not for homework), and Xbox/Play-Station/other electronic games that you play when sitting. This same question was asked for weekends. For each sedentary behavior an “hours per week” variable was created by multiplying the weekday hours per day by 5 and adding it to the weekend hours per day multiplied by 2. Students who reported 5+ hours of use were coded as having 6 hours. Total sedentary behavior per week was calculated as the sum of the three individual behaviors per week (Test-retest r =0.78 [parent]; r = 0.86 [adolescent]).

### Control variables

Race/ethnicity, educational attainment, and age were assessed by self-report. *Parent and adolescent race/ethnicity* was assessed with one survey item: ‘Do you think of yourself as 1) white, 2) black or African-American, 3) Hispanic or Latino, 4) Asian-American, 5) Hawaiian or Pacific Islander, or 6) American Indian or Native American’ and respondents were asked to check all that apply. Participants who checked ‘white’ and another option were included in the other category. Those who checked two non-white options were categorized as ‘mixed/other race’. Additionally, those checking Hawaiian/Pacific Islander or Native American were also categorized as ‘mixed/other race’ due to their small numbers in this dataset. *Parent educational attainment* was assessed using the following question, “What is the highest level of education that you have completed?” Response options included: less than high school, high school/GED, vocational/technical/trade school, associate degree, bachelor degree, graduate or professional degree. *Parent and adolescent age* was calculated using self-reported birth date and survey completion date.

### Statistical analysis

Descriptive statistics for study variables included means and standard deviations for continuous variables and frequencies and percentages for categorical variables. To compare differences between resident parent and nonresident parent variables, t-tests were conducted for continuous variables including BMI, hours of weekly physical and sedentary activity, daily servings of fruits and vegetables, weekly frequency of fast food consumption, daily consumption of sugary beverages, weekly frequency of eating breakfast, and a Chi-square test for the binary variable dieting.

Continuous dependent variables (adolescent BMI z-scores, weekly hours of physical activity, weekly hours of sedentary activity, daily servings of fruits and vegetables, weekly frequency of fast food consumption, daily consumption of sugary beverages, and weekly frequency of eating breakfast) were modelled using separate linear regressions. The corresponding independent variables for both resident and nonresident parents were included in each model. Because parent BMI was measured differently from adolescent BMI (i.e., adolescent = BMI z-score), adolescent BMI z-score was estimated for a 1-unit change in parent BMI (kg/m^2^). Adolescent dieting, a dichotomous variable, was modeled using logistic regression with the corresponding dieting variables of both resident and nonresident parents. Adjusted probabilities of adolescent dieting were computed from the logistic regression for each level of dieting (yes/no) for both resident and nonresident parents. These adjusted probabilities were used to calculate risk differences and corresponding standard errors were estimated by Taylor-series expansion. F-tests were used to compare differences between resident and nonresident parent regression coefficients of independent variables.

To determine if there were differences in effects between the resident and nonresident parents’ independent variables and adolescent gender, tests of interactions were conducted by including interaction terms in all models. Statistically significant differences by adolescent gender were not found for both resident and nonresident independent variables, and main effects are reported only. All final models were adjusted for adolescent gender and race/ethnicity and resident and non-resident parent gender and educational attainment.

## Results

Descriptive results showed that nonresident and resident parents engaged in similar amounts of healthful and unhealthful behaviors including: weekly hours of sedentary behavior, fruit and vegetable intake, sugar-sweetened beverage intake, frequency of eating breakfast, and the proportion of parents engaging in dieting behaviors (Table 2). In addition, resident and nonresident parents had similar BMI values. The only health behavior for which resident and nonresident parents significantly differed was fast food intake, with nonresident parents consuming significantly more fast food per week (p =0.002; Table 2). Additionally, nonresident parents’ physical activity levels (5.5 hours/week) were slightly higher than resident parents’ physical activity levels, although this finding was marginally significant (4.6 hours/week; p = 0.052).

**Table 2:** **Paired** ***t*** -test and chi-squared comparisons between resident and nonresident parents’ BMI, physical activity and eating behaviors*

	N**	**Resident parent**	**Non-resident parent**	**p-value**
**BMI** $\frac{\boldsymbol{kg}}{\left({\boldsymbol{m}}^{\boldsymbol{2}}\right)}$	185	27.3 (26.5, 28.1)***	27.7 (27.0, 28.5)	0.39
**Physical activity (hrs/wk)**	198	4.6 (4.0, 5.2)	5.5 (4.8, 6.1)	0.05
**Sedentary activity (hrs/wk)**	197	15.4 (13.8, 17.0)	16.3 (14.8, 17.7)	0.31
**Fruits & Vegetables (servings/day)**	199	3.5 (3.2, 3.8)	3.2 (2.9, 3.5)	0.19
**Fast food consumption (# times/wk)**	200	1.5 (1.3, 1. 7)	2.0 (1.7, 2.2)	**0.00**
**Sugary beverages (#/day)**	200	0.6 (0.5, 0.7)	0.7 (0.6, 0.8)	0.10
**Breakfast (#/wk)**	199	4.4 (4.0, 4.8)	4.0 (3.7, 4.4)	0.11
**Dieting (Proportion)**	198	0.5 (0.4, 0.5)	0.4 (0.4, 0.5)	0.14

*Resident parent = lives with child ≥50% of the time; Nonresident parent = lives with child <50% of the time

**N represents total in paired *t*-test comparisons.

***Values presented as Mean (95% CI); Bolded p-value represent statistically significant difference between resident and nonresident parent’s behavior at p < 0.05.

### Associations between resident and nonresident parents’ weight and weight-related behaviors and adolescents’ weight and weight-related behaviors

Both resident and nonresident parents’ BMI were associated with adolescents’ BMI z-score (p <0.001; Table 3). Additionally, resident parents’ sugar-sweetened beverage consumption was associated with adolescent sugar-sweetened beverage consumption (p =0.001). Furthermore, resident parents’ fruit and vegetable intake was associated with adolescent fruit and vegetable intake (p =0.048). However, resident parents’ fast food consumption, breakfast frequency, physical activity, sedentary behavior and dieting were not significantly associated with adolescents’ same behaviors.

**Table 3:** Association between adolescent BMI percentile, physical activity and eating behaviors and resident* and nonresident parents’ BMI, physical activity and eating behaviors**

	N***	Resident parent****	**Non-resident parent**
**BMI (z-score)**	185	**0.04 (0.02, 0.07)** ^A*****^	**0.06 (0.04, 0.09)** ^**A**^
**Physical activity (hrs/wk)**	198	0.11 (−0.06, 0.27)^A^	0.13 (−0.01, 0.27)^A^
**Sedentary activity (hrs/wk)**	197	−0.13 (−0.48, 0.22)^A^	0.01 (−0.36, 0.38)^A^
**Fruits & Vegetables (servings/day)**	188	**0.14 (0.00, 0.29)** ^**A**^	0.06 (−0.09, 0.21)^A^
**Fast food consumption (# times/wk)**	199	0.05 (−0.10, 0.20)^A^	−0.01 (−0.13, 0.11)^A^
**Sugary beverages (#/day)**	191	**0.33 (0.13, 0.54)** ^**A**^	0.04 (−0.16, 0.24)^A^
**Breakfast (#/wk)**	199	0.09 (−0.07, 0.24)^A^	0.02 (−0.13, 0.17)^A^
**Dieting (Risk difference)**	197	0.02 (−0.12, 0.15)^A^	−0.02 (−0.16, 0.12)^A^

*Resident parent = lives with child ≥50% of the time; Nonresident parent = lives with child <50% of the time

**Models adjusted for child’s gender, race/ethnicity, and resident and non-resident parent’s gender and educational attainment.

***N represents total in final model without missing information on outcome, exposure, and confounders.

****Values presented as β (95% CI) = expected change in means for continuous variables and proportions for dieting of the adolescent for a 1 unit increase in parents’ variable and 95% confidence interval; bolded if p < 0.05.

*****Coefficients not sharing a letter are statistically significant at α =0.05.

Nonresident parents’ physical activity was marginally associated with adolescent physical activity (p = 0.067). However, nonresident parents’ fruit and vegetable intake, sugar-sweetened beverage consumption, breakfast frequency, fast food intake, sedentary behavior, and dieting were not significantly associated with adolescents’ same behaviors.

Although there were significant associations between resident parents’ weight, sugar-sweetened beverage consumption, and fruit and vegetable intake and adolescents’ weight, sugar-sweetened beverage consumption and fruit and vegetable intake, these associations did not significantly differ between resident and nonresident parents. For example, the beta estimate of 0.33 representing the association between resident parent’s sugar-sweetened beverage consumption and adolescent’s sugar-sweetened beverage consumption was not significantly different from the beta estimate of 0.04 representing the association between nonresident parent’s sugar-sweetened beverage consumption and adolescent’s sugar-sweetened beverage consumption.

## Discussion

Results from the current study both reinforce findings from previous studies and expand findings from prior research. The finding that both resident and nonresident parents’ BMI were significantly associated with adolescents’ BMI z-score corroborates previous research [31-33] that lends support to the hypothesis that weight status and obesity are correlated within family, due to both genetic and environmental factors.

Additionally, the findings that resident parents’ fruit and vegetable intake and sugar-sweetened beverage consumption (but not other weight-related behaviors such as fast food intake, breakfast frequency, dieting, physical activity and sedentary behavior) were associated with similar behaviors in their adolescent children both corroborate and expand upon earlier findings regarding primary caregiver’s influence on adolescents’ weight-related behaviors [4,34]. Specifically, previous studies have found mixed results regarding associations between primary parents’ modeling of fast food consumption, fruit and vegetable intake, sugar-sweetened beverage consumption, dieting behaviors, physical activity and sedentary behaviors and adolescents’ same behaviors [33,35-38]. The current study expands previous research suggesting that resident parents’, but not nonresident parents’, fruit and vegetable intake and sugar-sweetened beverage consumption, may influence adolescents’ likelihood of engaging in the same behaviors. One potential implication of these findings, although results are not consistent across all adolescent weight-related behaviors, is that interventions may be able to solely target resident parents’ fruit and vegetable intake and sugar-sweetened beverage consumption in order to influence adolescents’ engaging in these key behaviors that have been strongly linked to obesity, weight gain (sugar-sweetened beverage intake) [39] or weight loss/maintenance overtime (fruit and vegetable intake) [28]. For example, adolescent obesity prevention interventions may want to focus on emphasizing resident parent modeling of healthful eating behaviors and parenting practices, above and beyond nonresident parent eating behaviors.

Additionally, nonresident parent’s physical activity was marginally associated with adolescent’s physical activity and thus may be a potential influence on adolescents engaging in physical activity themselves. This is an interesting finding that needs to be replicated in future research, due to the marginal significance of the finding. It may be important to consider intervening on resident parent’s influence on adolescent’s dietary intake and nonresident parent’s influence on adolescent’s physical activity in adolescent obesity interventions. One potential hypothesis for this finding is that when children visit their nonresident parent they may spend more time being active together, thus providing an opportunity for the nonresident parent to model physical activity and in turn, increase the likelihood that the adolescent will be more physically active overall. Future research is needed to understand this finding, such as qualitative research that can explore reasons why this association may exist.

Study strengths and limitations should be taken into account when interpreting the study findings. The current study had several strengths. This analysis was connected to a data set that includes a large, diverse, population-based sample with a high response rate, allowing for more confidence in generalization of findings to similar populations. In addition, independent reports were collected from resident parents, nonresident parents and adolescent themselves, which is not commonly done. Furthermore, several weight-related behaviors were measured (e.g., fruit and vegetable intake, breakfast frequency, sugar-sweetened beverage intake, fast food consumption, physical activity, sedentary behavior, dieting) and adjustments were made for possible third variable confounding of results (age, parent education, race/ethnicity, parent BMI). Although information was collected on a large number of adolescents, the number of adolescents in the current study was limited to much smaller subset of the overall population. This reduced size resulted in larger standard errors and less precise associations. Furthermore, this study was cross-sectional. Because we were unable to examine longitudinal associations, we cannot establish temporality of associations. For example, it may be the case that adolescents’ weight and weight-related behaviors influence parents’ weight and weight-related behaviors rather than parents’ being more influential. Most likely, the influence is bi-directional and it is important for future studies to use mixed-methods studies to identify parent and adolescent opinions about the bi-directional influential patterns around weight and weight-related behaviors that may be occurring in the home environment. In addition, longitudinal studies are needed to examine bi-directional influences of weight and weight-related behaviors. Furthermore, all survey measures were self-report measures and may have been prone to social desirable responses from both parent and adolescent participants.

## Conclusions

Resident parents’ BMI, fruit and vegetable intake and sugar-sweetened beverage consumption was significantly associated with similar behaviors in their adolescent children, however only nonresident parents’ BMI and physical activity (marginal) was associated with adolescents’ BMI and physical activity. Thus, future research is needed to further explore the association between resident and nonresident parent weight and weight-related behaviors and adolescents’ weight and weight-related behaviors. Additionally, it would be important for future research to examine resident and nonresident parental influences on younger children’s weight and weight-related behaviors, who may be more dependent on their caregivers’ modeling of weight and weight-related behaviors in the home environment. Furthermore, it may be useful for healthcare providers to ask adolescents about both resident and non-resident parents’ eating and physical activity behaviors, how they spend time with each parent, and suit recommendations to individual adolescent-parent pair.
